# Solitary fibrous tumor of the prostate: a case report and the systematic review of 74 published cases

**DOI:** 10.3389/fonc.2025.1593184

**Published:** 2025-09-26

**Authors:** Hang Zhang, Chunyue Yan, Daowen Zhang, Zhipeng Chen, Kun Wang, Fan Zhou, Ming Yang, Fei Wang

**Affiliations:** ^1^ Department of Radiology, Luzhou People’s Hospital, Luzhou, China; ^2^ Department of Emergency Medicine, Luzhou People’s Hospital, Luzhou, China

**Keywords:** solitary fibrous tumor, prostate, systematic review, follow up, rare

## Abstract

**Purpose:**

This study aimed to report a rare case of solitary fibrous tumor of prostate (pSFT) and achieve the first systematic review of 74 published pSFT cases, and to integrate the end-to-end management of pSFT from preoperative diagnosis, treatment, and follow-up.

**Methods and case report:**

This study reports a additional case of pSFT. Subsequently, we performed a systematic review of PubMed, Web of Science, Cochrane Library, and Embase databases for potentially relevant articles on pSFT from inception of database to October 2024. Two researchers independently screened eligible literature, extracted data, and summarized data.

**Results:**

A 71-year-old male patient presented to our hospital with a three-month history of dysuria, and prostate-specific antigen (PSA) was within the normal range. After transurethral prostatectomy, the histopathological diagnosis was pSFT. During three years of outpatient follow-up, the patient had no recurrence. Ultimately, forty literatures were included with 74 cases of pSFT, and age ranged from 21 to 89 years (mean 57.5 years, median 61.5 years). There were 42 cases (56.8%) in North America, 22 cases (29.7%) in Asia, 9 cases (12.2%) in Europe, and 1 case (1.4%) in Oceania. The PSA values were within the normal range in almost all cases, and about 47.3% (35/74) cases underwent ultrasound, magnetic resonance imaging or CT examination before surgery. Notably, the most characteristic imaging biomarkers of the tumor were continuous and gradual enhancement from the periphery to the center with visible separation and capsule. The malignancy rate was 21.6% (16/74). There was no significant difference between benign and malignant pSFT of the lesion size (P > 0.05). Meanwhile, STAT6 and/or NAB2-STAT6 fusion genes were very sensitive biomarkers for pSFT. The median follow-up time of 38 patients with pSFT was 18 months (2–168 months), and the disease-free survival of benign, borderline and malignant pSFT was statistically significant (P=0.011).

**Conclusions:**

This study presents a new case and provides the first systematic review for 74 cases of pSFT, integrating clinical presentation, pathology, imaging, and follow-up data to assist surgeons in surgical planning. Surgical resection is the preferred treatment for pSFT, and regular follow-up is of vital importance. Due to the heterogeneity among current studies, future research may require standardized data reports and the establishment of a homogeneous public database to prepare for subsequent pSFT risk stratification studies.

**Systematic review registration:**

https://www.crd.york.ac.uk/prospero/, identifier CRD420251004804.

## 1 Introduction

Solitary fibrous tumor (SFT) is a relatively rare stromal spindle cell neoplasm, most of which are benign, and a few are malignant (1–3). Previous studies show that patients with SFT of the prostate (pSFT) have a wide range of ages, and the exact pathogenic cause of pSFT is still unknown (4–8). Most cases with SFT of the prostate choose surgical resection, however, some SFT cases exhibit aggressive biological behavior after operation (4–8). The diagnostic criteria of malignant SFT have not yet been clarified, but the invasive pathological features are obvious (9). In recent years, pSFT has only been described in individual cases or a few case reports. However, there is a lack of systematic review on the pathogenesis, distribution characteristics, clinical manifestations, imaging findings and postoperative prognosis of pSFT. Meanwhile, there are differences in the diagnosis and treatment of some case reports, and there is a lack of objective evaluation of large samples, which makes a challenge of clinical diagnosis and comprehensive postoperative follow-up (7, 8).

Presently, some studies have found a strong correlation between pSFT and NAB2-STAT6 fusion gene, which may be the key to changing the difficult situation of diagnosis (10, 11), and pSFT is extremely rare with 21.6% malignant cases and 23.0% misdiagnosis rate. Clinical diagnosis and treatment of pSFT are still problems which need to be solved urgently. Interestingly, the countries that published pSFT cases were mainly concentrated in the United States, Japan, and China (4, 11). Yasumichi Takeuchi et al. summarized 25 pSFT cases and found that NAB2-STAT6 could help distinguish pSFT from stromal tumor of uncertain malignant potential (11). Yueqiang Peng et al. summarized 39 pSFT cases and reported the first case of micro pSFT (4). Micro pSFT of the prostate can not be detected by imaging examination but can be easily distinguished from benign prostatic hyperplasia using histopathology and immunohistochemistry. Additionally, Yoichiro Okubo et al. (8) summarized the immunohistochemical results of 34 pSFT cases and found that only CD34 and progesterone receptor (PR) were positive, which may lead to missed diagnosis of pSFT. Marcal LP et al. (12) reported the imaging findings of 13 pSFT cases, suggesting that preoperative imaging findings can accurately determine the origin and extent of tumor invasion, and play a pivotal role in diagnosis and staging. Moreover, Yuemei Xu et al. summarized the pathological features of 3 micro pSFT cases and 12 pSFT, and found that signal transducer and activator of transcription 6 (STAT6), PR and Ki-67 may help distinguish micro pSFT, STUMP and prostatic stromal sarcoma (PSS) (13). Similarly, Bakhshwin A et al. (14) achieved the experience in the treatment of 4 pSFT and found that the use of GATA binding protein 3 (GATA3) and PR expression levels and monoclonal STAT6 immunohistochemical analysis helped differentiate pSFT from PSS. However, most studies of pSFT are reported by individual cases or several case reports with great inter-individual differences. The clinical pathological diagnosis, immunotherapy and follow-up are still controversial, and there is still no comprehensive and clear diagnosis and follow-up protocol. Moreover, previously reported reviews are plagued by a lack of data.

As far as we know, this is the first comprehensive systematic review and objective evaluation of the pathogenesis, distribution characteristics, clinical manifestations, imaging findings and postoperative prognosis of pSFT. Therefore, we aimed to report an additional case of pSFT and achieve the first comprehensive systematic review of 74 published pSFT cases, and to integrate the end-to-end management of pSFT from preoperative diagnosis, treatment, and follow-up. These findings may provide assistance for clinical individualized diagnosis, treatment and follow-up decision-making.

## 2 Materials and methods

### 2.1 Case report

A 71-year-old male patient was admitted to the hospital due to dysuria for more than 3 months and condition worsened for one day. Before admission, the patient had no obvious hematuria, abdominal pain, palpitation, chest tightness and other discomfort. After completing the relevant preoperative examinations, surgical treatment was performed and the tumor was pathologically confirmed pSFT. Postoperative outpatient follow-up was performed for 3 years with no signs of recurrence. Our study was approved by the Medical Ethics Committee of Luzhou People’s Hospital and received informed consent from patients.

### 2.2 Systematic review

#### 2.2.1 Retrieval Methods

**Figure 1:**
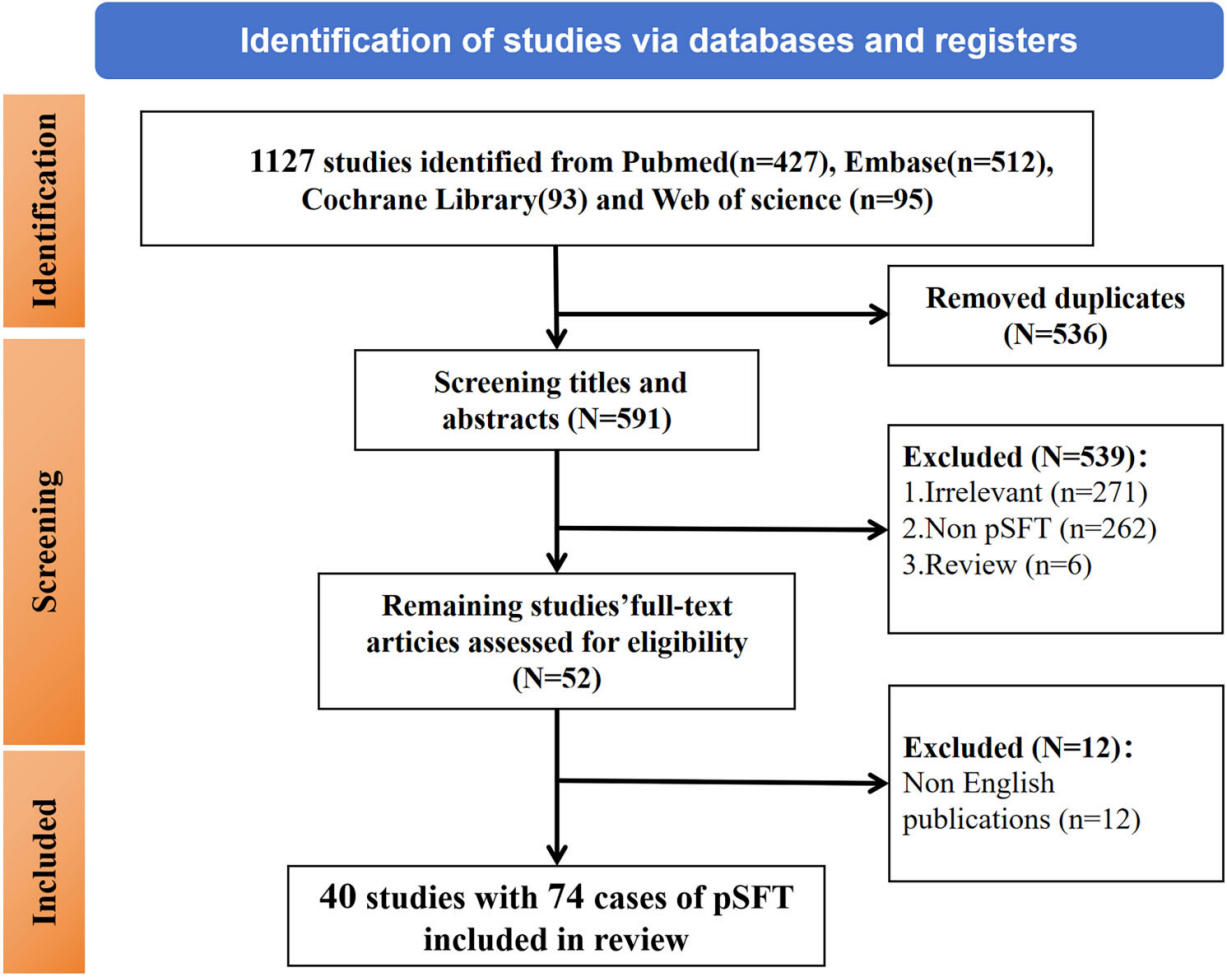
A flowchart of the literature screening process.

This systematic review adheres to the PRISMA guidelines, and our review has pre-registered on PROSPERO (CRD420251004804). We performed a systematic search of PubMed, Web of Science, Cochrane Library, and Embase databases for potentially relevant articles on pSFT from inception of database to October 2024. The search terms were “solitary fibrous tumor” AND “prostate” OR “prostatic” AND “mesenchymal tumor or cancer”. And a secondary search through references was also performed. The inclusion criteria were pathologically confirmed primary solitary fibrous tumor of the prostate from published English literature. The following exclusion criteria were adopted (1): republished literature (2); unclear pathological diagnosis (3); review or conference abstracts. Ultimately, a flowchart of the literature screening process was summarized in **Figure 1**.

#### 2.2.2 Literature screening and data extraction

The retrieved literatures were imported into NoteExpress. After removing duplicates, HZ and CYY (Six and five years in abdominal diagnostics) independently extracted the basic characteristics of the studies according to the predefined inclusion and exclusion criteria, and FW (Nine years in abdominal diagnostics) and MY (27 years in abdominal diagnostics) reviewed and validated the obtained data. The basic characteristics of the literatures included the first author, publication year, cases of pSFT, lesion size, the operational approach and the postoperative follow-up time, etc. Moreover, The classification of benign, borderline, or malignant for pSFT was based on the original definitions provided in the included studies. The obtained data were reviewed three times.

#### 2.2.3 Quality assessment

Given that most included studies were case reports, we qualitatively evaluated their methodological quality using the JBI Critical Appraisal Checklist for Case Reports. Due to limited data, no quantitative scoring was performed, but studies lacking histopathological confirmation or follow-up data were excluded.

### 2.3 Objectives

**Figure 2:**
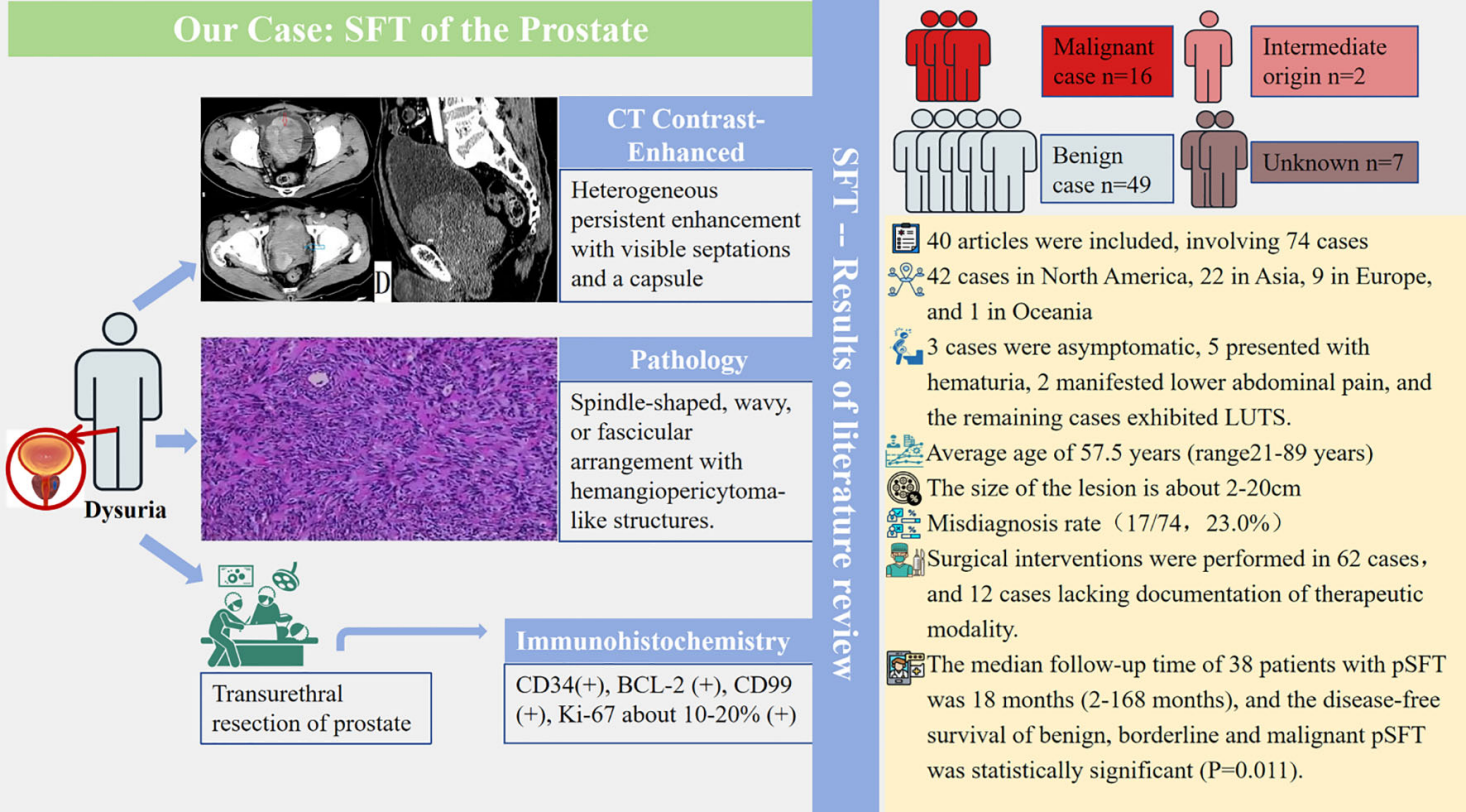
The main working flowchart of this study.

This study aims to present a case of our pSFT and conduct a comprehensive and systematic review and synthesis of the pathogenesis, distribution characteristics, clinical manifestations, imaging findings, and postoperative prognosis of pSFT, and the flowchart of this study is summarized in **Figure 2**. Furthermore, despite its rarity, this research expands the sample size through a systematic review approach, integrating end-to-end management of pSFT from preoperative diagnosis, treatment, to follow-up.

## 3 Results

### 3.1 Case report

**Figure 3:**
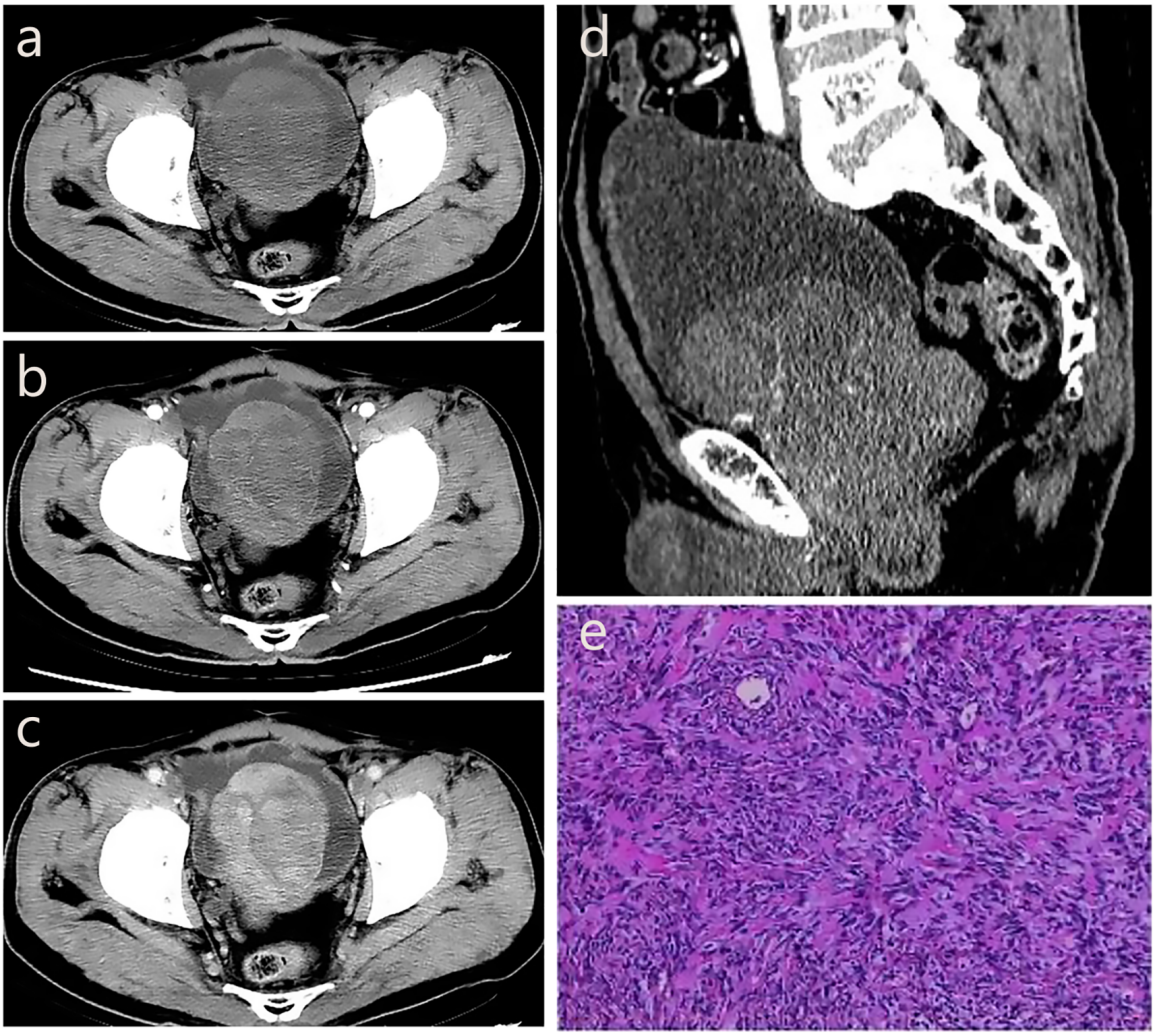
The CT imaging biomarkers and pathological of our pSFT. **(a, d)** CT shows an increase in prostate volume, with a mass of approximately 4.5cm × 5.8cm protruding into the bladder, with clear boundaries and low density. **(b)** CT enhanced scan shows mild enhancement in the arterial phase, which is flocculent and uneven. **(c)** In the venous phase, the tumor shows continuous and gradual enhancement from the periphery to the center, with visible separation and capsule. **(e)** Pathological examination showed a spindle cell tumor of the prostate, and the tumor cells were spindle-shaped, wavy, storiform or fascicular arrangement, and hemangiopericytoma-like structure could be seen and nuclear division was clear to see, spindle-shaped or oval.

A 71-year-old male patient was admitted to the hospital due to dysuria for more than 3 months and condition worsened for one day. Before admission, the patient had no obvious hematuria, abdominal pain, palpitation, chest tightness and other discomfort. Laboratory tests showed that prostate-specific antigen (PSA=1.99 ng/mL) and free prostate-specific antigen (f-PSA=0.18 ng/mL) were within the normal range. Urine routine tests showed elevated levels of urinary protein (Pro=2g/L), urinary occult blood (BLD=3), and urinary white blood cells (WBC-F=40.6 cells/μL). CT suggested an increase in prostate volume, with a mass of approximately 4.5 cm×5.8 cm protruding into the bladder, with clear boundaries and low density. CT enhanced scan achieved mild enhancement, which is flocculent and uneven. In the venous phase, the tumor indicated continuous and gradual enhancement from the periphery to the center, with visible separation and capsule (**Figures 3a–d**).

The patient underwent transurethral resection of the prostate (TURP) under spinal anesthesia 6 days after admission. During the operation, the bilateral lobes of the prostate were obviously hyperplasia into the cavity, the posterior lip of the bladder neck was moderately elevated, and a round mass with a size of about 4cm×5cm was seen at about 11 o’clock of the prostate projecting into the urethra. Postoperative pathological examination showed a spindle cell tumor of the prostate, and the tumor cells were spindle-shaped, wavy, storiform or fascicular arrangement, and hemangiopericytoma-like structure could be seen and nuclear division was clear to see, spindle-shaped or oval (**Figure 3e**). The immunohistochemistry results showed CK (–), P63 (–), P504S (–), S100 (–), CD34 (+), BCL-2 (+), CD99 (+), SMA (–), Desmin (–), 10%-20% Ki-67-positive. The result of pathological diagnosis was pSFT. Notably, the patient was followed up in the outpatient department for 3 years after surgery, and there were no signs of recurrence.

### 3.2 Results of systematic review

#### 3.2.1 Literature screening and basic characteristics

Ultimately, forty literatures (3–9, 11–43) were included with 74 cases of pSFT, and age ranged from 21 to 89 years (mean 57.5 years, median 61.5 years). The clinical manifestations depend on the size of the tumor and whether the tumor involves surrounding organs. Specifically, three patients were asymptomatic, five patients had hematuria, two patients had lower abdominal pain, and the remaining patients had mild or severe lower urinary tract symptoms. Except for 3 pSFT cases with slightly increasing PSA levels, the PSA values of other cases (including our case report) were in the normal range. The basic characteristics of pSFT are shown in **Table 1**.

**Table 1:** The basic characteristics of 75 cases of prostate SFT.

First author/year of publication	Age	Imaging examination	Symptoms	Tumor size (cm)	PSA (ng/ml)	Treatment	Follow-up	Relapse/metastasis/death
Yasumichi Takeuchi/2021 (11)	43	MRI	No symptom	3×3.4	0.675	RP	2 years	No
João Matos/2020 (19)	66	MRI	Urinary frequency, urgency	NA	0.4	RP	5 years	No
Andrea Ronchi/2017 (24)	62	CT	LUTs and constipation	20×10	5.8	RP	8 years	No
Anamika Mishra/2020 (25)	28	MRI, CT	Dysuria	5.8×6.4×6.5	Normal	Enucleation	14 years	No
Nilay Nishith/2020 (30)	54	US, CT	Urinary frequency, urgency	5×4×3	Mildly elevated	RP	Regular follow-up	No
Ya-Ting Liu/2019 (7)	46	MRI, US, CT	No symptom	6×7×6	Normal	RP	5 years	Relapse
Yueqiang Peng/2022 (4)	50	MRI, US, CT	LUTs and nocturia	Small	0.64	RP	3 months	No
Brent Gilbert/2020 (29)	78	MRI, CT	Constipation and lower abdominal pain	6.3×4.6	2	Enucleation	12 months	No
Ming Zhao/2022 (31)	68	MRI	No symptom	4	NA	NA	12 months	No
Soma Osamu/2017 (20)	65	US, CT	Nocturia	10	0.92	RP	18 months	No
Yuemei Xu/2021 (13)	64	MRI	NA	7.6×4.5	NA	RP	6 months	No
	50			19×11	NA	Enucleation	7 years	No
	57			18×10	NA	Enucleation	NA	NA
Alejandro Hevia Feliu/2022 (16)	85	MRI	LUTs and dysuria	17×12×6	NA	Enucleation	18 months	No
Qiang Cheng/2019 (18)	43	MRI, US	Hematuria	8×4.5×3.5	0.686	RP	3 months	No
Michael R. Pins/2001 (32)	57	CT	No symptom	10×7×7	1.2	RP	15 months	No
	73	CT	Dysuria, urinary frequency and urgency	6×5	3.5	RP	21 months	No
Mehrnaz Gharaee-Kermani/2014 (33)	53	NA	Hematuria and uroschesis	NA	NA	RP	NA	NA
Yoshikazu Tanaka/2018 (27)	68	MRI, US, CT	Hematuria and urinary frequency	6×5	Normal	Enucleation	54 months	Relapse with metastasis
H Sekine/2001 (34)	42	MRI, US, CT	Dysuria	NA	1.1	RP	18 months	No
Wenyan Yang/2015 (35)	46	MRI, US	LUTs and dysuria	6.4×5.6×5.7	0.68	RP	18 months	No
Tomomoto Ishikawa/2004 (36)	64	US, CT	Dysuria and hematuria	12.5×9.5×8.3	Normal	RP	3 months	No
Taketsugu Ishii/2004 (37)	36	MRI, US	Dysuria	NA	1.6	TURP (partial)	6 years	No
YukioTakeshima/1997 (38)	42	MRI, CT	Dysuria, constipation and uroschesis	14×13×11	NA	RP	10 months	No
Heidi Talvitie/2011 (5)	66	MRI, US	Dysuria, rectal and lower urinary tract symptoms	4.2×3×5	Slightly elevated	RP	a short period of time	No
	69	NA	LUTs, uroschesis and hematuria	5	NA	TURP		No
Laurence Moureau-Zabotto/2012 (6)	60	MRI, US, CT	Dysuria, urinary frequency and urgency	15×11.5×9	Normal	RCP	28 months	No
Yoichiro Okubo/2020 (8)	40	CT	Lower urinary tract symptoms	6×5×4	NA	Enucleation	6 months	No
W H Westra/2000 (28)	65	MRI	Uroschesis	11	NA	RCP	2 months	Metastasis
Ahmed Bakhshwin/2020 (14)	73	NA	Symptoms of benign prostatic hyperplasia (3)/hematuria (1)	5	NA	TURP	NA	NA
	49			9		(1)TURP,	14 months	Metastasis
						(2)RP		
	55			8		(1)TURP, (2)RCP	30 months	Relapse
	69			13		(1)TURP, (2)RCP	12 months	No
Sota Oguro/2006 (17)	35	MRI, US	Uroschesis	5.2×5	1.22	Enucleation	12 months	Relapse
D.Parada Domínguez/2010 (39)	65	US	Dysuria	8×6.5×2.4	7.52	RP	NA	NA
M. Grasso/2002 (26)	21	US	Dysuria and uroschesis	2.4	NA	Enucleation	NA	NA
Puneet Bhargava/2012 (40)	37	MRI, US	Uroschesis	10.5×8.5	NA	NA	NA	NA
Peng Luo/2020 (3)	47	NA	Dysuria	8	NA	RP	9 months	No
Aleksandar Vodovnik/2005 (41)	87	US	LUTs and hematuria	2~9	Normal	RP	NA	Died the first day after surgery
Balagopal Nair/2007 (42)	37	MRI	LUTs	10	NA	RP	2 years	No
Andrea B Galosi/2009 (43)	60	MRI, US, CT	LUTs	8×7×6	0.6	RP	6 months	No
Leonardo P. Marcal /2022 (12)	50	CT	NA	NA	NA	NA	NA	NA
	40	MRI						NA
Masanori Noguchi/2002 (15)	46	US, CT	No symptom	22×17×16	1.2	Enucleation	14 months	No
Yuliang Sun/2003 (23)	56	NA	NA	NA	NA	NA	NA	NA
Mehsati Herawi/2007 (9)	Median: 65, range	NA	All have varying degrees of LUTs	Median: 10.5 (8.5–15)	NA	TURP (1); RP (4 cases); RCP (2 cases); pelvic exenteration (2 cases); enucleation (1 case)	NA (4), 7 months(1), 1 to 10 years(5)	NA (4 cases); death (1 case); Relapse (5 cases)
	46-75 (10 cases)							
Gunes Guner/2016 (22)	Median: 61.5, range	NA	NA	NA	NA	TURP (6 cases); RP (2 cases); biopsy (4 cases)	NA	NA
	42-89 (12 cases)							
Roni M Cox/2020 (21)	NA(7 cases)	NA	NA	NA	NA	NA	NA	NA
Our case	71	CT	Dysuria	4×5	1.99	TURP	3 years	No

MRI, magnetic resonance imaging; US, ultrasound; CT, computed tomography; LUTs, lower urinary tract symptoms; PSA, prostate-specific antigen; RP, radical prostatectomy; RCP, radical cystoprostatectomy; TURP, transurethral resection of the prostate; NA, not available; pSFT, solitary fibrous tumor of the prostate.

#### 3.2.2 Regional distribution and lesion size

**Figure 4:**
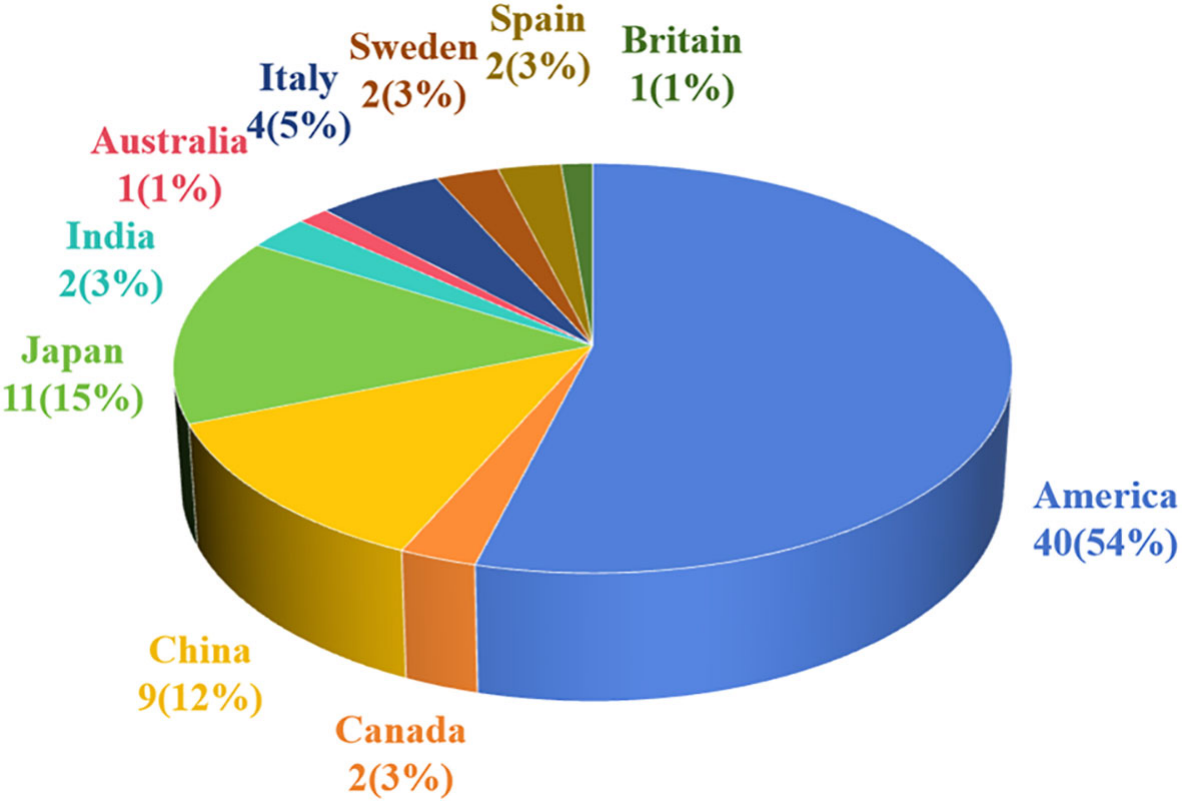
The regional distribution plot of pSFT.

Notably, 74 cases of pSFT have been reported, including 42 cases (56.8%) in North America, 22 cases (29.7%) in Asia, 9 cases (12.2%) in Europe, and 1 case (1.4%) in Oceania. The regional distribution was shown in **Figure 4**. Additionally, pSFT varies in size (2–20 cm) and most reported cases are larger than 5 cm. Moreover, according to the statistics of retrieved literature, there were 2 cases of pSFT with intermediate origin and 7 cases of pSFT with unknown benign or malignant. On the one hand, the reported size of benign cases of pSFT were about 30/49 (61.2%), with an average size of 8.9 cm. On the other hand, the reported size of malignant cases of pSFT were about 15/16 (94.0%), with an average size of 11.3 cm. There was no significant difference between benign and malignant pSFT regarding lesion size (P > 0.05).

#### 3.2.3 Inspection methods

In our case, a plain CT scan and enhanced CT scan were used to assist in the localization and diagnosis of the tumor. In 74 cases of pSFT reported, about 35/74 (47.3%) cases underwent ultrasound (US), magnetic resonance imaging (MRI) or CT examination before operation. Among them, 23/35 (65.7%) underwent MRI examination, 17/35 (48.6%) underwent CT examination and 16/35 (45.7%) underwent US examination.

#### 3.2.4 Pathology and immunohistochemistry

The pathological and immunohistochemical data of the reported cases were shown in **Table 2**. BCL-2 (31/31, 100%), CD34 (53/55, 96.4%) and STAT6 (34/35, 97.1%) displayed the highest positive expression rates. Followed by CD99 (21/24, 87.5%) and PR (8/15, 53.3%), Ki-67 positive index was 85.2% (range 0-20), including 6 cases of PR focal expression, 1 case of Ki-67 positive index >50%. At last, the positive rates of CD117, smooth muscle actin (SMA), S100 protein and desmin were very low.

**Table 2:** The immunohistochemical staining of 74 cases of pSFT.

IHC staining	Positive rate	Reported cases	Unreported cases
CD34	96.4% (53/55)	55	19
Bcl-2	100% (31/31)	31	43
Ki-67 (n=27)	mean Ki-67 labeling index (n=23) was 85.2% (range, 0–20%)	27	47
	mean Ki-67 labeling index (n=3) was 11.1% (range,20–50%)		
	Ki-67 labeling index (n=1) was > 50%		
STAT6	97.1% (34/35)	35	39
CD117	11.8% (2/17)	17	57
CD99	87.5% (21/24)	24	50
SMA	6.45% (2/31)	31	43
S100	3.7% (1/27)	27	47
Desmin	10.7% (3/28)	28	46
PR	53.3% (8/15), Focally+ (6)	15	59

CD34, cluster differentiation 34; Bcl-2, B-cell lymphoma-2; STAT6, signal transducer and activator of transcription 6; CD117, cluster differentiation 117; CD99, cluster differentiation 99; SMA, smooth muscle actin; PR, progesterone receptor; pSFT, solitary fibrous tumor of the prostate.

#### 3.2.5 Operational approach and benign or malignant of pSFT

TURP was performed in our case. Among the 74 cases, 12 cases had unknown treatment (12/74, 16.2%), and 4 (4/74, 5.4%) cases received biopsy for diagnosis. Other 62 cases of pSFT were all treated by surgery, including radical prostatectomy (29/62, 46.8%), radical cystoprostatectomy (6/62, 9.7%), TURP (10/62, 16.1%), and enucleation (including partial enucleation) (13/62, 21.0%). Furthermore, 5 cases underwent two operations and one case underwent three operations, and 11 cases (11/74, 14.9%) received postoperative radiotherapy or chemotherapy. For invasiveness, 16 cases (16/74, 21.6%) of pSFT were malignant, and 56 cases (56/74, 75.7%) of pSFT were benign and 2 cases (2/74, 2.7%) of pSFT were borderline.

#### 3.2.6 Follow-up

**Figure 5:**
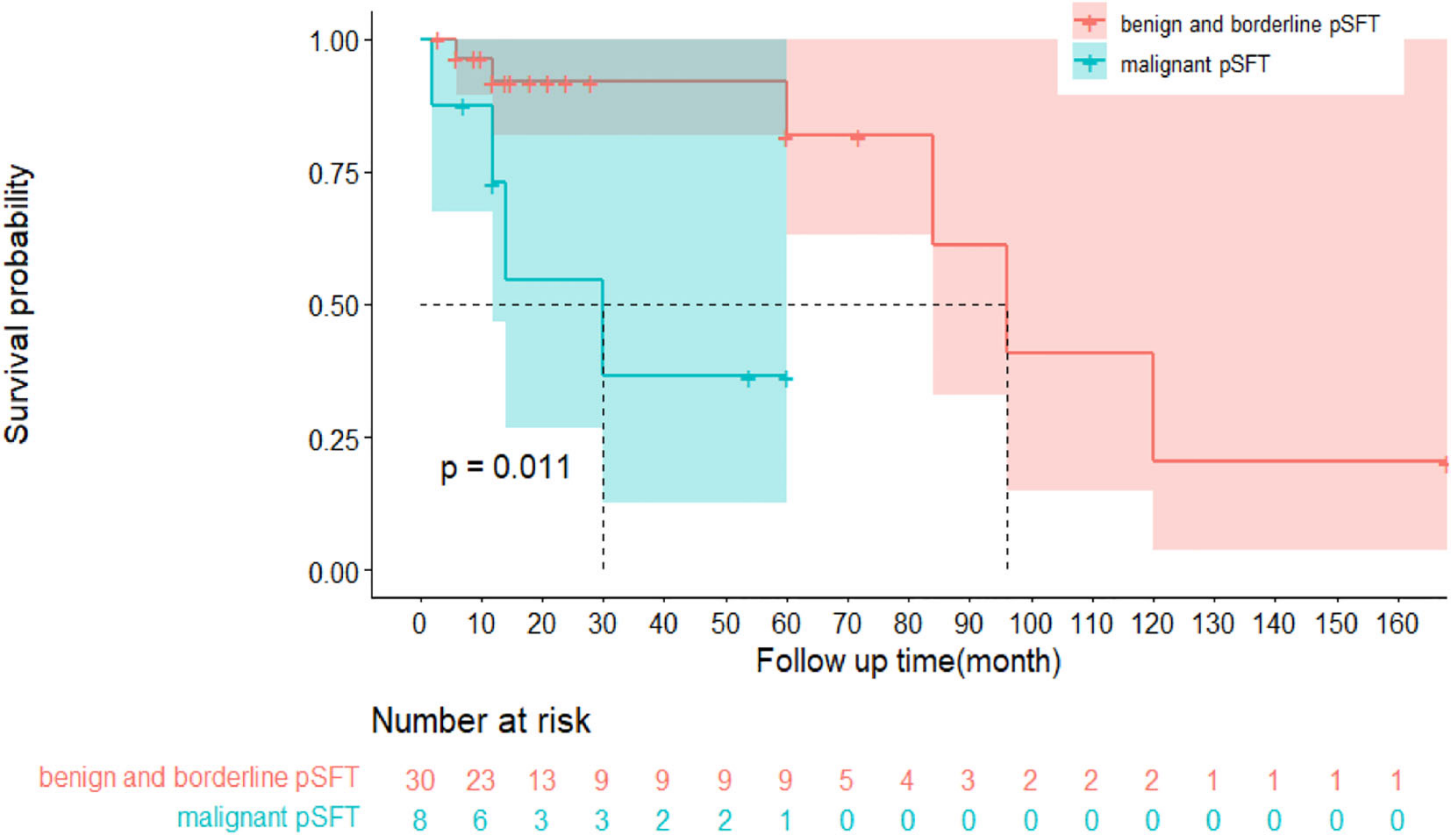
Kaplan-Meier survival curve plot of disease-free survival (DFS) between benign and borderline pSFT and malignant pSFT.

Notably, of the 74 pSFT cases, only 38 pSFT cases had follow-up records and follow-up time, and the follow-up time range was 2 to 168 months. To be more specific, 26 cases of pSFT were benign, 10 cases of pSFT were malignant, and 2 cases of pSFT were borderline. Importantly, **Figure 5** suggested that there was significant difference of disease-free survival (DFS) between benign and borderline and malignant tumors (P=0.011, Log-rank test). However, due to incomplete time-to-event data, hazard ratios could not be calculated reliably.

## 4 Discussion

To the best of our knowledge, this study is the first to objectively and systematically evaluate the regional distribution, size, inspection methods, immunohistochemistry, operation and follow-up of 75 cases of pSFT. Notably, the most characteristic imaging biomarkers of pSFT were continuous and gradual enhancement from the periphery to the center with visible separation and capsule (12, 17). Moreover, although pSFT is very extremely rare, this study innovatively expanded the sample size through a systematic review approach, integrating end-to-end management of pSFT from preoperative imaging diagnosis and treatment to follow-up. Ultimately, these findings may provide a new vision for clinical diagnosis, treatment, and follow-up of pSFT.

### 4.1 New perspectives in imaging diagnosis: analysis of various imaging features of pSFT

To better differentiate pSFT from prostate cancer (PCa) and benign prostatic hyperplasia (BPH), and refer to previous studies (6, 7, 11–13, 16–20, 40, 44–49), the key imaging features are summarized in **Table 3**. According to included literature, MRI is more preferred in recent years, which has the characteristics of clear differentiation of soft tissue structures, high resolution and multi-sequence imaging analysis. While US examination is mainly used for the initial screening of lesions and localization for biopsy site. The most characteristic sign of enhanced CT scan is the continuous and gradual enhancement of the tumor from the periphery to the center, with visible separation and capsule, which may be related to the fact that the SFT of the prostate is mainly composed of tumor spindle cells with hemangiopericytoma-like structure. Since pSFT lacks specific clinical symptoms, comprehensive preoperative imaging examinations, including US, MRI, and CT, allow for an accurate assessment of the tumor’s origin, size, morphology, and its relationship with adjacent structures (6, 12, 17). Additionally, high-resolution and small-field MRI can clearly delineate the prostate mass and its boundaries with adjacent structures, such as the bladder wall and rectal layers. This imaging technique is crucial for surgical approach planning.

**Table 3:** Comparative Imaging Features of pSFT, PCa, and BPH.

Imaging Modality	pSFT	PCa	BPH
T_1_-weighted MRI	Iso- to slightly hypointense	Iso- to hypointense (especially in peripheral zone)	Isointense
T_2_-weighted MRI	Heterogeneous, often low signal due to collagen	Hypointense in peripheral zone	Heterogeneous, nodular, mixed signal
DWI/ADC	Mildly hyperintense on DWI; variable ADC	Markedly hyperintense on DWI; low ADC	Minimal restriction or none
DCE-MRI	Gradual, delayed enhancement from periphery to center	Early rapid enhancement with washout	Mild or heterogeneous enhancement
Capsule/Margin	Often encapsulated, well-circumscribed	Ill-defined, infiltrative margins	Well-defined
Location	Anywhere in prostate, often large and lobulated	Usually in peripheral zone	Mainly in transition zone
Mass effect	Common, displacing adjacent organs	May invade adjacent structures	None or mild
PSMA PET/CT	Rarely positive; non-specific uptake	Strong uptake in high-grade tumors	Typically negative

DWI, diffusion-weighted imaging; ADC, apparent diffusion coefficient; DCE, dynamic contrast-enhanced; PSMA, prostate-specific membrane antigen; PCa, prostate cancer; BPH, benign prostatic hyperplasia.

In terms of imaging characteristics, pSFT typically exhibits low-intensity signals on T_1_-weighted sequences and heterogeneous, mixed signals on T_2_-weighted sequences (12). These signal features are associated with factors such as the collagen fiber content of the tumor, as well as the presence of degeneration or necrosis. In most cases, T_2_-weighted images predominantly show low signal intensity (11, 13, 16). Additionally, in T_1_-weighted contrast-enhanced and T_2_-weighted images, SFT typically appears as a multilocular, encapsulated heterogeneous mass with significant enhancement in its solid components. On multiphase dynamic MRI, the tumor demonstrates a delayed filling pattern, with gradual enhancement progressing from the periphery to the center (6, 12, 17–19). On diffusion-weighted imaging (DWI), the tumor region typically exhibits mildly high signal intensity (7, 13). Although MRI contrast-enhanced scanning was not performed in this case, the tumor’s composition and enhancement pattern align with findings from previous studies (6, 12, 17–19). Furthermore, nuclear medicine studies have demonstrated that PSMA PET/CT has high specificity for early diagnosis and staging of prostate cancer (44). However, in some cases, PSMA PET/CT scans have detected intense uptake not only in the prostate tissue but also in the pleura and humerus, which was later pathologically confirmed to be SFT (45, 46). Although reports suggest PSMA-PET/CT may identify SFTs, its diagnostic value in pSFT remains unvalidated and should be interpreted with caution. More evidence is needed before recommending PSMA-based imaging in routine pSFT workup. In addition, when the tumor’s blood supply is unclear, DSA can serve as an auxiliary diagnostic tool (20). Imaging biomarkers play an important supplementary role in the diagnosis of prostate SFT, providing critical information for preoperative clinical decision-making. However, due to the nonspecific imaging features of pSFT and their overlap with other prostate tumors, the final diagnosis still relies on histopathological and immunohistochemical characteristics (10, 13, 14).

### 4.2 Unveiling the truth: key strategies to overcome the diagnostic dilemma of pSFT

Statistical data suggested that the misdiagnosis rate of pSFT was approximately 23.0%. Among these, 82.4% (14/17) of misdiagnosed cases were incorrectly identified as prostatic hyperplasia or stromal tumors. Additionally, in some cases, tumors from other pelvic regions were misdiagnosed as pSFT (6, 50, 51). This misdiagnosis phenomenon was closely related to the unique anatomical location and clinical presentation of pSFT. Similarly, pSFT typically arose as abnormal hyperplasia, with lower urinary tract symptoms (e.g., difficulty urinating, frequent urination) as the primary clinical manifestations (4, 8, 11). Histologically, pSFT overlaps with other prostatic lesions characterized by spindle cell morphology (25). Although markers such as CD34, Bcl-2, and CD99 were highly expressed in SFT, they were not specific to the tumor, which increases the risk of pathological misdiagnosis. For pathologists, accurately differentiating pSFT from other prostatic stromal tumors remains a significant challenge.

Refer to previous studies (4, 8, 52–54), **Table 4** summarized immunohistochemical markers of pSFT, BPH and pCa. Over 95% of SFT cases exhibit the NAB2-STAT6 gene fusion characteristic. Therefore, positive STAT6 immunohistochemical staining and/or detection of the NAB2-STAT6 gene fusion can significantly enhance the accuracy and consistency of SFT diagnosis. Currently, the STAT6 gene and its immunohistochemical detection are recognized as highly sensitive and specific markers for the diagnosis of SFT (9–11, 55). Early studies reported that STAT6 expression might occur in cases of prostatic stromal hyperplasia, but recent studies (14, 21, 22, 56, 57) have demonstrated that this expression is absent when detected with monoclonal STAT6 antibodies. The effectiveness of monoclonal STAT6 antibodies has been validated, showing higher sensitivity and specificity in distinguishing pSFT from prostatic stromal hyperplasia. Additionally, Bakhshwin A (14) reported findings from the treatment of four cases of mSFT, highlighting that the expression of GATA3 and PR, along with monoclonal STAT6 immunoexpression, may aid in distinguishing mSFT. Our study revealed that approximately 53.3% (8/15) of pSFT cases exhibited PR expression. However, this finding differs from Bakhshwin A’s report, suggesting that PR expression may also occur in benign SFT. Additionally, when STAT6 testing fails to provide a definitive diagnosis, pathologists are advised to perform a comprehensive analysis using additional immunohistochemical markers. Studies on aldehyde dehydrogenase 1 (ALDH1) have demonstrated that combining STAT6 and ALDH1 staining techniques can significantly enhance the specificity of prostate SFT diagnosis (22, 58). This approach has garnered increasing attention and application in recent studies. Interestingly, two cases of prostate SFT reported in previous literature (6, 9) unexpectedly revealed focal areas of prostate adenocarcinoma. This finding suggests that clinicians may perform a more comprehensive differential diagnosis when evaluating pSFT to avoid overlooking potential coexisting lesions, thereby enhancing diagnostic accuracy and reliability.

**Table 4:** Immunohistochemical Profiles of pSFT Compared to BPH and PCa.

Markers	pSFT	BPH	PCa
CD34	Strongly positive (96.4%)	Focal or weakly positive	Negative
STAT6	Strong nuclear positivity (~97%)	Negative	Negative
Bcl-2	Strongly positive	Negative	Variable
CD99	Moderate positivity (87.5%)	Negative	Negative
PR	Positive (53.3%)	Positive	Negative or focal
Ki-67	Usually <20%, rare >50%	Very low	Variable, often high in high-grade
SMA	Negative or focal	Positive in stroma	Negative
Desmin	Negative or focal	Positive in stroma	Negative
PSA	Negative	Negative	Strongly positive
AMACR (P504S)	Negative	Negative	Positive
P63/CK5/6	Negative	Positive (basal cells)	Negative
S100	Rare/focal (3.7%)	Negative	Negative

Positive, Immunoreactive; Negative, No staining; Focal, Scattered positive areas.

CD34, cluster differentiation 34; Bcl-2, B-cell lymphoma-2; CD99, cluster differentiation 99; PR, progesterone receptor; SMA, smooth muscle actin; STAT6, signal transducer and activator of transcription 6; AMACR, alpha-methylacyl-CoA racemase; PCa, prostate cancer; BPH, benign prostatic hyperplasia.

### 4.3 Enhanced risk identification: multidimensional exploration of diagnosis and prognostic assessment in prostatic mSFT

Research on prostate mSFT indicated that the proportion of malignant cases was approximately 21.6%, nearly twice the rate reported in previous studies (4). In the evaluation of pSFT malignancy, we observed that the predictive value of the Ki-67 positivity index, an indicator of proliferative activity, remains controversial (23, 59). According to the literature (23), the mean Ki-67 index is approximately 1.9% in benign SFT and around 6.11% in mSFT. Another study (59) suggested that when the Ki-67 positivity index exceeds 20%, there is a possibility of malignancy in renal SFT. However, in our study, the patient’s Ki-67 positivity index ranged between 10% and 20%, with no apparent signs of malignancy before surgery, and no recurrence observed during a 3-year follow-up. Furthermore, some mSFT cases have Ki-67 indices below 20% (13), while certain benign SFT cases exhibit Ki-67 indices exceeding 20% (9). Among the 74 cases analyzed, more than half had no recorded Ki-67 positivity index. Therefore, the current data is insufficient to support predicting SFT malignancy based solely on the Ki-67 index percentage, and the large-scale, multicenter studies are required to further validate the reliability and applicability of this indicator in the future. Demicco et al. (60) proposed a risk stratification model that incorporates factors such as patient age, tumor size, and mitotic activity. These factors were strongly associated with the risk of SFT metastasis or patient mortality. Subsequent studies (61) confirmed the effectiveness of this model in preoperative risk assessment and suggested that it could serve as a valuable reference for preoperative biopsy. However, Andrea Ronchi et al. (24), in a long-term follow-up of high-risk mSFT cases, found that some patients categorized as highest risk did not develop recurrence or metastasis even after 8 years. This suggests that the biological behavior of pSFT remains somewhat unpredictable, warranting further studies for ongoing monitoring.

Notably, dedifferentiated SFT shares similar histological and immunohistochemical features with prostatic sarcomatoid carcinoma, making misdiagnosis likely (61). The use of STAT6 immunohistochemistry and/or NAB2-STAT6 gene fusion testing, in combination with multiple immunohistochemical markers and enhanced imaging studies, can significantly improve diagnostic accuracy. Other studies (62, 63) have reported that some SFTs can secrete large amounts of insulin-like growth factor 2 (IGF-2), leading to paraneoplastic hypoglycemia syndrome in patients. Therefore, in the management of SFT patients, attention should be given to potential metabolic complications and the risk of malignant progression. In summary, the diagnosis and prognostic assessment of prostate mSFT require the integration of multiple factors, including the Ki-67 index, risk stratification models, immunohistochemical testing, and clinical follow-up data. Future research may aim to refine diagnostic criteria and risk prediction tools to improve the management and therapeutic outcomes of mSFT.

### 4.4 Surgery first, monitoring essential: exploring treatment and follow-up strategies for prostate SFT

Currently, surgical resection is the preferred and most effective treatment for pSFT (4, 8). However, treatment plans should be tailored to the individual patient’s condition, particularly for younger patients, where management strategies must balance functional preservation and long-term prognosis. In previous reports (25, 26), two cases of pSFT in young patients were documented. One case, reported by Anamika Mishra, showed no recurrence after a 14-year follow-up, while the other had no detailed follow-up information available. However, the risk of pSFT recurrence does not always correlate with pathological features. In some cases, despite negative surgical margins and the absence of histological signs of aggressiveness, local recurrence occurred postoperatively (7, 27). Conversely, some high-risk cases with aggressive features showed no recurrence during long-term follow-up (5, 6, 13, 24), though this may be attributed to insufficient follow-up duration. Therefore, for pSFT, complete surgical resection is crucial to prevent recurrence, even in the absence of overt malignant features (27). For large, extensively invasive tumors, en bloc resection involving adjacent organs may be necessary to achieve complete clearance of the lesion (9, 20, 28, 50). In terms of radiotherapy and chemotherapy, existing literature (20, 30, 64) indicates that SFT shows low sensitivity to both treatments. Therefore, for advanced or metastatic cases where complete resection is not feasible, more effective treatment options need to be explored. Some studies (28, 51, 65) have shown that for patients with residual or recurrent lesions, radiotherapy or chemotherapy can serve as adjunctive treatment options. However, routine adjuvant therapy is not currently recommended for pSFT cases with complete surgical resection. For patients with unresectable or metastatic disease, systemic therapy—primarily involving chemotherapy and anti-angiogenic agents—has shown positive outcomes in certain cases (4, 16, 51). However, due to the small sample size of pSFT patients who received postoperative adjuvant therapy (only 11 cases, accounting for 14.9%), a comprehensive evaluation of prognosis is currently not feasible. Therefore, further research is needed to substantiate the efficacy of radiotherapy and chemotherapy.

Notably, among 74 analyzed cases, the recurrence or metastasis rate of pSFT was 8.1%, which is consistent with reports in the literature (11, 51). Studies have shown that mSFT is more prone to recurrence or metastasis than benign SFT (4, 14). However, some mSFT cases showed no recurrence during long-term follow-up, which may be attributed to insufficient follow-up duration. Interestingly, some benign SFT cases exhibited a trend of increasing lesion diameter during multiple preoperative examinations (16, 27, 29). Although histologically benign, the biological behavior may indicate latent malignant potential, further highlighting the uncertainty of SFT malignancy and prognosis. Given the risk of recurrence and late-stage metastasis in pSFT, early surgical intervention is recommended when clinically feasible to minimize the likelihood of recurrence. Postoperative monitoring should be rigorous, and for patients with residual lesions or metastatic risk, adjuvant chemotherapy or radiotherapy may be necessary (3, 47, 66). Additionally, previous research (4, 25, 51) indicated that SFT might have a prolonged recurrence period and the potential for late-stage recurrence. Given the unpredictable nature of recurrence, annual imaging is recommended within 5 years. For patients with high-risk histologic features, prolonged or lifelong follow-up may be advisable.

### 4.5 Limitations and challenges of the study

The limitations of this study are summarized as follows: (1) Insufficient immunohistological data: Among the 74 analyzed cases, the data of markers such as Ki-67, PR and CD117 were incomplete. In addition to NAB2-STAT6, the sample size should be expanded in the future to standardize the pathological diagnosis of pSFT. (2) The patients exhibited a wide age range and significant individual differences. However, the study did not categorize or analyze symptom characteristics across different age groups, limiting the depth of disease feature analysis; Furthermore, apart from the patient’s age, the number of samples with common characteristics was small, which was insufficient to conduct univariate and multivariate analyses to identify independent risk factors affecting the survival prognosis of pSFT. In the future, perhaps the collection and management of pSFT should be standardized, and a homogeneous public database should be established to prepare for subsequent research on the risk stratification of pSFT. (3) The study reported a relatively high proportion of lost-to-follow-up cases (approximately 38.7%), which limited the ability to draw comprehensive conclusions on the prognosis of pSFT patients and may have affected the reliability of prognostic analysis. Future research requires standardized data reports and the establishment of an open-source follow-up management mechanism to ensure the completeness of pSFT samples.

## 5 Conclusions

Prostate SFT is extremely rare, and complete clinical treatment and follow-up records are even scarcer. This study presents a new case and provides the first systematic review of the diagnostic and therapeutic process for 74 cases of pSFT, integrating clinical presentation, pathology, imaging, and follow-up data to assist surgeons in surgical planning. Importantly, surgical resection is the preferred treatment for pSFT, but regular postoperative follow-up is crucial. Future research may require standardized data reports and the establishment of an open-source follow-up management mechanism to ensure the completeness of pSFT samples; Moreover, it might be necessary to establish a homogeneous public database to prepare for the subsequent pSFT risk stratification research.

## Data Availability

The original contributions presented in the study are included in the article/supplementary material. Further inquiries can be directed to the corresponding author.
